# Silicon Accumulation and Photosynthetic Capacity of *Dendrocalamus brandisii* in Response to Sodium Silicate Foliar Application Across Vegetative Phenological Stages

**DOI:** 10.3390/plants14172624

**Published:** 2025-08-23

**Authors:** Yuntao Yang, Lei Huang, Lixia Yu, Fangwei Zhu, Ju Chang, Maobiao Li, Shuguang Wang, Changming Wang, Hui Zhan

**Affiliations:** 1 Key Laboratory for Forest Resources Conservation and Utilization in the Southwest Mountains of China, Ministry of Education, Kunming 650224, China; yangyuntao@swfu.edu.cn (Y.Y.); leihuang@swfu.edu.cn (L.H.); yulixia@swfu.edu.cn (L.Y.); zhufw3397@163.com (F.Z.); 13984722084@163.com (J.C.); stevenwang1979@126.com (S.W.); wcm@swfu.edu.cn (C.W.); 2 College of Forestry, Southwest Forestry University, Kunming 650224, China; benjiamin@swfu.edu.cn

**Keywords:** silicon, phenological stages, photosynthesis, photoassimilate accumulation, *Dendrocalamus brandisii*

## Abstract

Silicon plays a positive role in plant growth and physiological activities; however, silicon fertilizer application in bamboo remains limited. This study explored the silicon accumulation and photosynthetic capacity of *Dendrocalamus brandisii* in response to sodium silicate (SS) foliar application across vegetative phenological stages. The results showed that August (shooting stage) and May (branching and leafing stage) were the critical periods for silicon accumulation. SS significantly enhanced the net photosynthetic rate (Pn), chlorophyll content, and photosystem activity (Fv/Fm, Fv′/Fm′), particularly in August and May. Correlation analysis revealed that silicon content was significantly positively correlated with photosynthetic parameters (Pn, chlorophyll a/b) and photoassimilate accumulation (soluble sugar, starch), confirming that silicon optimized leaf light capture and carbon assimilation capacity by promoting phytolith formation. This research provides a theoretical foundation for the application of silicon fertilizers in bamboo forest cultivation.

## 1. Introduction

Silicon is a mineral element essential for most plant growth [1]. However, most silicon in soil cannot be absorbed by plants, and only a very small amount of monosilicic acid [Si(OH)_4_] can be absorbed and utilized by plants. Silicon can be used as a biostimulant in actual production and applied to plants by foliar spraying, incorporation into soil, or irrigation fertilization [2]. Numerous studies have shown that silicon accumulation correlates closely with photosynthesis, benefiting plant growth and physiological activities [3]. Silicon enhances the mechanical strength of plant tissues. For example, the application of nano-silicon fertilizer improves the lodging resistance of wheat stems, significantly enhancing wheat quality [4]. The accumulation of silicon significantly improves the mechanical stability of plants, as they exhibit better structural support performance than carbon-based compounds [5]. This contributes to the development of erect and robust defensive structures such as stems, branches, and leaves through complex biochemical pathways [6]. Enhancing silicon absorption and accumulation can boost nutrient uptake (N, P, K), elevate chlorophyll concentration, and improve leaf arrangement, so as to optimize light interception and photosynthetic rates in crops like rice, wheat, sugarcane, and banana [7,8,9,10]. During the rice growth and reproduction stages, silicon application enhances photosynthetic rates, delays chlorophyll decline, and increases leaf thickness, chloroplast surface area, and mesophyll cell wall thickness. It also boosts mesophyll and stomatal conductivity, reduces intercellular CO_2_ concentration, and improves overall leaf photosynthetic function [11,12]. However, knowledge of the silicon applications in bamboo forests remains limited.

Bamboo is one type of plant richest in silicon, with its epidermal tissue containing up to 70% organic silicon [13]. There were significant differences in silicon content among different tissues and organs of the same bamboo species [14]. Bamboo leaves had the greatest silicon content [3]. With leaf maturation, silicon deposition in leaves increased [15]. Additionally, Motomura et al. [16] found that silicification on the *Sasa veitchii* leaves of different ages was not the same. Short cells in epidermal cells were preferentially silicified, while the number of silicified cells in other epidermal cells gradually increased with leaf aging. There was a certain difference in silicification among cells of different growth stages. Silicon in *Dendrocalamus giganteus* leaves was highest at the late shooting stage and lowest at the branching and leafing stage [17]. Previous research on Bambusoideae plants focused on the content of silicon and morphology of phytoliths. However, silicon accumulation and its physiological role on photosynthesis at different phenological stages in response to silicon application remains unclear.

*Dendrocalamus brandisii*, a sympodial bamboo species, is native to tropical and subtropical regions of Southeast Asia, with its distribution encompassing China (particularly southern and southwestern Yunnan), Myanmar, Laos, Vietnam, and Thailand [18]. In China, *D. brandisii* acreage is among the largest forest plantations in southern and southwestern Yunnan. *D. brandisi* exhibits a short growth cycle and vigorous regeneration capacity, making it an excellent woody biomass alternative. Its shoots are edible with rich nutrients, making it an excellent dual-purpose bamboo for both shoots and culms. Increasing efforts are being made to utilize *D. brandisii* resources. This study analyzed the impact of foliar sodium silicate application on the silicon accumulation and photosynthesis of *D. brandisii* leaves at different phenological stages, e.g., shooting stage (August), late shooting stage (November), dormancy stage (January), and branching and leafing stage (May), to provide a theoretical basis for revealing the difference of silicon accumulation in different phenological stages and elucidating its physiological functions. This research also aimed to offer a theoretical foundation for the application of silicon fertilizers in bamboo forest cultivation.

## 2. Results

### 2.1. Silicon Content in D. brandisii Leaves Under Treatment at Different Phenological Stages

**Figure 1:**
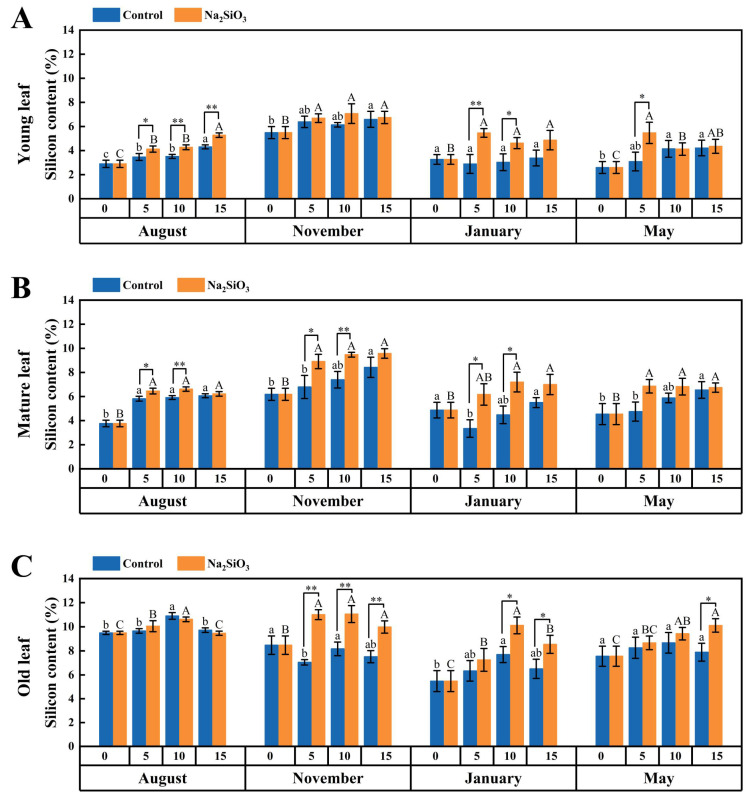
Silicon content in *D. brandisii* leaves at different phenological stages under control and addition of SS (%). (**A**) Young leaf. (**B**) Mature leaf. (**C**) Old leaf. Data are presented as mean ± standard deviation. Different uppercase letters above trend lines denote the significant differences between leaves at different stages in the control group at *p* < 0.05 according to LSD. Different lowercase letters above trend lines denote significant differences between leaves at different stages in the experimental group at *p* < 0.05 according to LSD. One asterisk denotes significant differences at *p* < 0.05 and two asterisks at *p* < 0.01 between leaves on the same day (Similarly hereinafter).

It was observed that the silicon content in *D. brandisii* young and mature leaves in the control group increased from August to November, indicating that bamboo accumulated a significant amount of silicon during the shooting stage. However, in January, a significant decrease in the young and mature leaves was observed. In May, the silicon content in *D. brandisii* leaves showed a slightly increasing trend. It was noted that old leaves had higher silicon content than young and mature leaves (Figure 1).

After SS treatment, the silicon content in *D. brandisii* leaves of different ages further increased with time compared to the control. During the shooting stage in August, silicon content in young and mature leaves increased significantly, particularly in young leaves, which continued to increase until the 15th day (Figure 1A). In the late shooting stage in November, silicon content in mature and old leaves enhanced significantly with SS treatment. It was observed that the silicon in old leaves increased more obviously and reached a maximum value of 11.05% on the 10th day (Figure 1C). During the dormancy stage in January, although the overall silicon content was low, it increased with SS treatment; specifically on the 5th and 10th day, silicon content in young and mature leaves showed a significantly increase. *D. brandisii* began branching and leafing in May. The silicon content in young and old leaves showed a further increment on the 5th and 15th day, respectively (Figure 1).

### 2.2. Net Photosynthetic Rate (Pn) of D. brandisii Leaves Under Treatment at Different Phenological Stages

**Figure 2:**
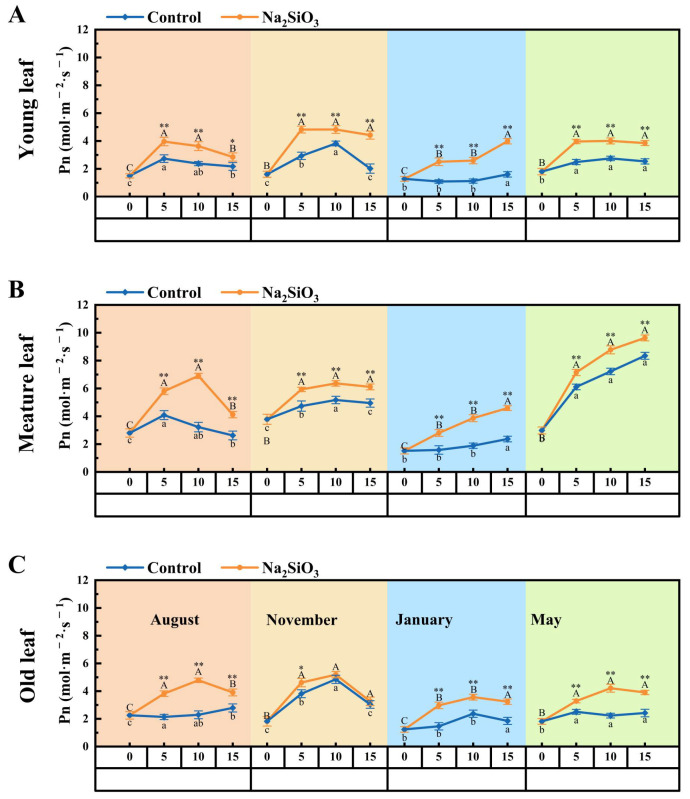
Net photosynthetic rate (Pn) of *D. brandisii* leaves at different phenological stages under control and addition of SS (mol·m^−2^·s^−1^). (**A**) Young leaf. (**B**) Mature leaf. (**C**) Old leaf.

The net photosynthetic rate (Pn) of *D. brandisii* leaves in the control group was observed to slightly increase from the shooting stage in August to the bamboo late shooting stage in November, and significantly decreased during the dormancy stage in January. Then a substantial increase was observed in branching and leafing stage in May, reaching a peak of 9.63 mol·m^−2^·s^−1^ on the 15th day in mature leaves. It was noted that the Pn varied in the order of mature leaf > young leaf > old leaf (Figure 2).

After SS treatment, the Pn of *D. brandisii* leaves of different ages at different phenological stages showed varying degrees of increase over time. In August, the Pn of leaves of different ages was significantly further enhanced, particularly the young and mature leaves. When *D. brandisii* entered the dormancy stage in January, the overall Pn was lower, but the Pn of leaves of different ages treated with SS was significantly greater than that of the control. In May, which was the branching and leafing stage for *D. brandisii*, the Pn of leaves of different ages with SS treatment still showed a higher level compared to the control (Figure 2).

### 2.3. Chlorophyll Fluorescence Rate in D. brandisii Leaves Under Treatment at Different Phenological Stages

**Figure 3:**
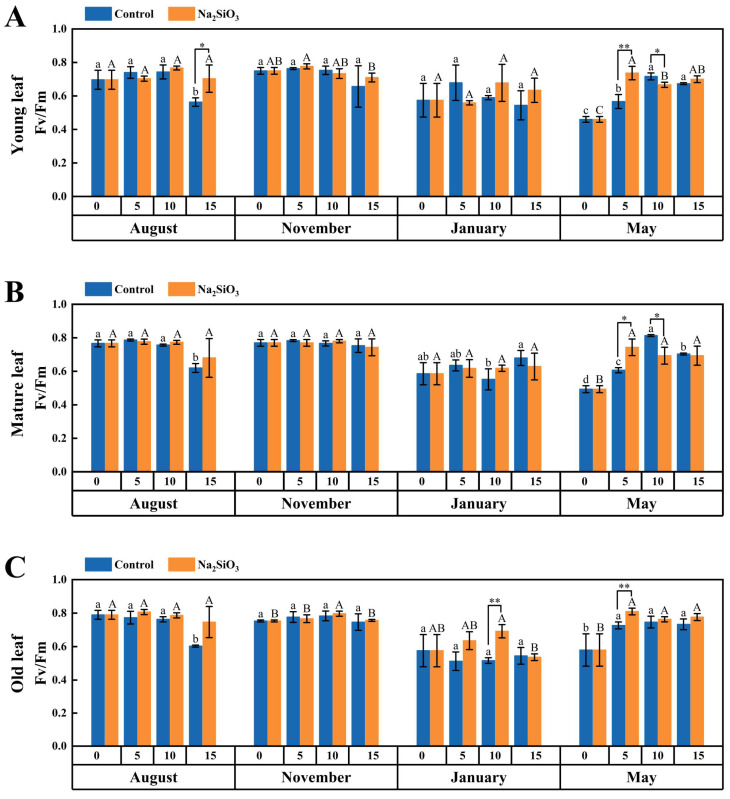
Fv/Fm rate of *D. brandisii* leaves at different phenological stages under control and addition of SS. (**A**) Young leaf. (**B**) Mature leaf. (**C**) Old leaf.

For the control group, the Fv/Fm of *D. brandisii* leaves of different ages at different phenological stages remained stable from August to November, but showed a downward trend in January of dormancy stage. When *D. brandisii* entered the branching and leafing stage in May, the Fv/Fm rate gradually recovered (Figure 3).

After SS application, Fv/Fm of *D. brandisii* leaves at different phenological stages enhanced. In August, Fv/Fm of young leaves significantly enhanced specifically on the 15th day. However, in November, no obvious enhancement in Fv/Fm rate of leaves was observed. In January, although the overall Fv/Fm values were low, with SS application, Fv/Fm of increased to some extent, with a significant increase observed in old leaves on the 10th day. When *D. brandisii* entered the branching and leafing stage, the Fv/Fm rate gradually recovered, and after SS application, Fv/Fm further improved on the 5th day, with the most significant increase in young leaves (Figure 3).

**Figure 4:**
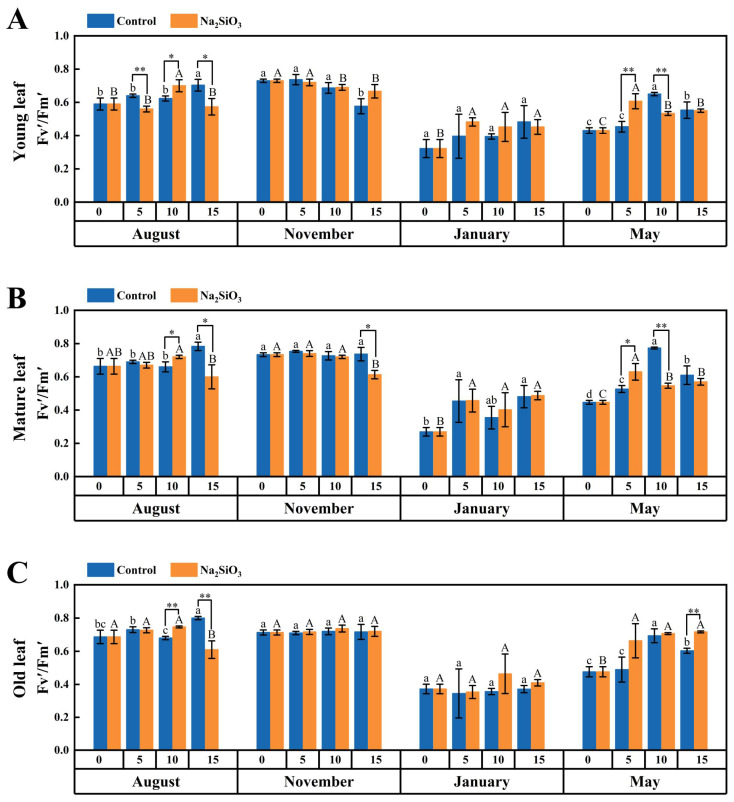
Fv′/Fm′ rate of *D. brandisii* leaves at different phenological stages under control and addition of SS. (**A**) Young leaf. (**B**) Mature leaf. (**C**) Old leaf.

It was noted that the Fv′/Fm′ rate of *D. brandisii* demonstrated a similar trend to Fv/Fm, which showed a stable trend from the shooting stage in August to November, but decreased in January when *D. brandisii* entered the dormancy stage. A significant rise in May of the branching and leafing stage was observed (Figure 4).

With SS treatment, the Fv′/Fm′ rate of *D. brandisii* leaves at different phenological stages increased. In August, the Fv′/Fm′ of leaves of different ages further increased significantly on the 10th day. However, SS application did not enhance Fv′/Fm′ rate significantly in November. When *D. brandisii* entered the dormancy stage, the overall Fv′/Fm′ rates were relatively low. While SS treatment had a certain promoting effect, the effects were not significant. In May, the Fv′/Fm′ of young and mature leaves was significantly enhanced by SS treatment on the 5th day, while Fv′/Fm′ of old leaves was further enhanced until the 15th day (Figure 4).

### 2.4. Photosynthetic Pigments of D. brandisii Leaves under Treatment at Different Phenological Stages

#### 2.4.1. Chlorophyll a

**Figure 5:**
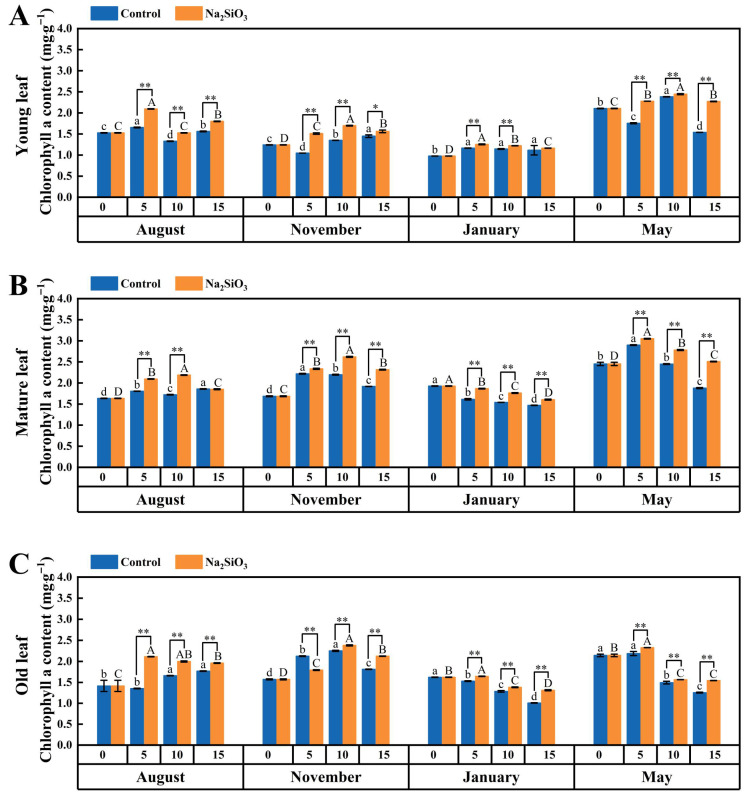
Chlorophyll a content in *D. brandisii* leaves at different phenological stages under control and addition of SS. (**A**) Young leaf. (**B**) Mature leaf. (**C**) Old leaf.

It was found that chlorophyll a increased from August to November, but decreased significantly in January, and tended to increase again in May. The chlorophyll a content was greater in the mature leaves than in young and old leaves (Figure 5).

With SS application, the chlorophyll a content in *D. brandisii* leaves increased at different phenological stages. In August, the chlorophyll a content in leaves of different ages significantly enhanced on the 5th and 10th day, and SS showed positive effect in old leaves until the 15th day. In November, SS treatment demonstrated a pronounced effect in young and mature leaves during 15th days, but for the old leaves, the enhancement was observed until the 10th day. In the dormancy stage in January, the enhancement in chlorophyll a was observed in *D. brandisii* leaves of different ages on the 5th and 10th day, but on the 15th day, the chlorophyll a enhancement in young leaves was not obvious. In the branching and leafing stage in May, the chlorophyll a in leaves of different ages was significantly promoted at the time of SS application (Figure 5).

#### 2.4.2. Chlorophyll b

**Figure 6:**
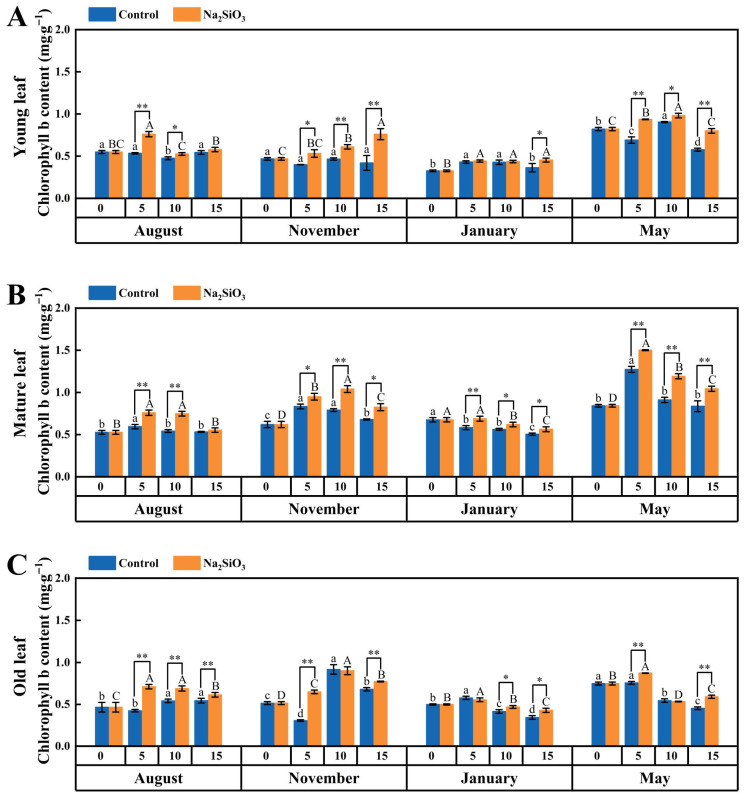
Chlorophyll b content in *D. brandisii* leaves at different phenological stages under control and addition of SS. (**A**) Young leaf. (**B**) Mature leaf. (**C**) Old leaf.

Similarly to the trend of chlorophyll a, chlorophyll b in *D. brandisii* leaves of different ages showed increasing trend starting August, followed by a decrease in January and then slightly increased again in May (Figure 6).

With SS application, the chlorophyll b content in *D. brandisii* leaves also increased at different phenological stages. In August, chlorophyll b in *D. brandisii* leaves of different of ages further increased, particularly the enhancement effect in the old leaves lasted until the 15th day. In November, the chlorophyll b content in the young and mature leaves increased with time of SS application, reaching a peak on the 15th and 10th day, respectively. The chlorophyll b in old leaves also showed enhancement, particularly on the 5th and 15th day. During the dormancy stage in January, the chlorophyll b in the mature leaves further increased on the 5th day and the enhancement lasted until the 15th day, while chlorophyll b in young leaves did not significantly enhance until the 15th day. In May, the chlorophyll b in the young and mature leaves continued to increase until the 15th day, whereas the old leaves showed significant increases on the 5th and 15th days (Figure 6).

#### 2.4.3. Total Chlorophyll

**Figure 7:**
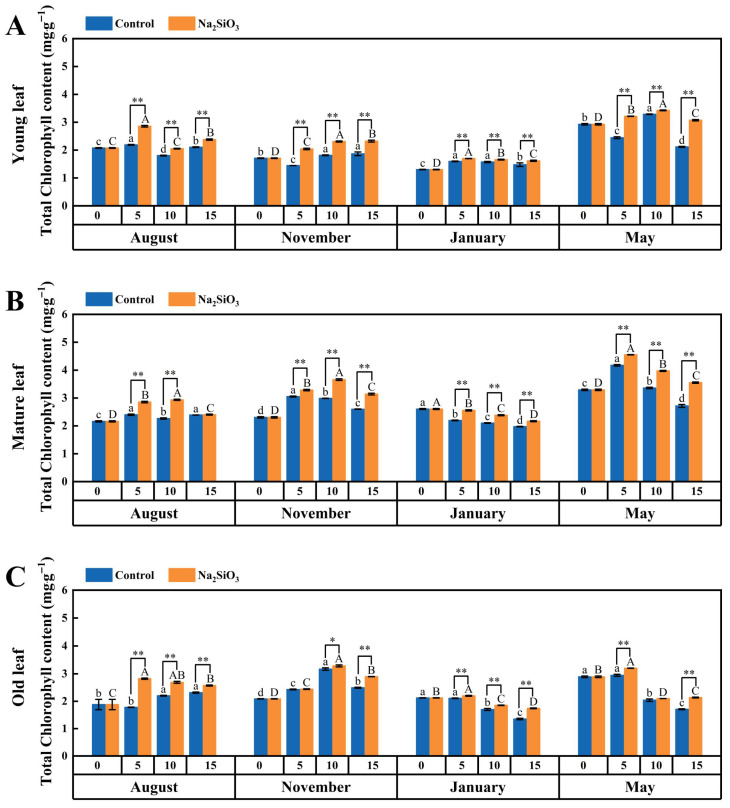
Total chlorophyll in *D. brandisii* leaves at different phenological stages under control and addition of SS. (**A**) Young leaf. (**B**) Mature leaf. (**C**) Old leaf.

It was observed that the total chlorophyll of the control showed similar trends to chlorophyll a and b (Figure 7).

After SS treatment, the total chlorophyll content in *D. brandisii* leaves of various ages increased significantly, especially in the young leaves, where the enhancement was particularly noticeable across different phenological stages. In contrast, mature leaves showed no clear increase in total chlorophyll on May’s 15th day, and in older leaves, the effect was not significant on either November’s 5th day or May’s 10th day. (Figure 7).

#### 2.4.4. Carotenoid

**Figure 8:**
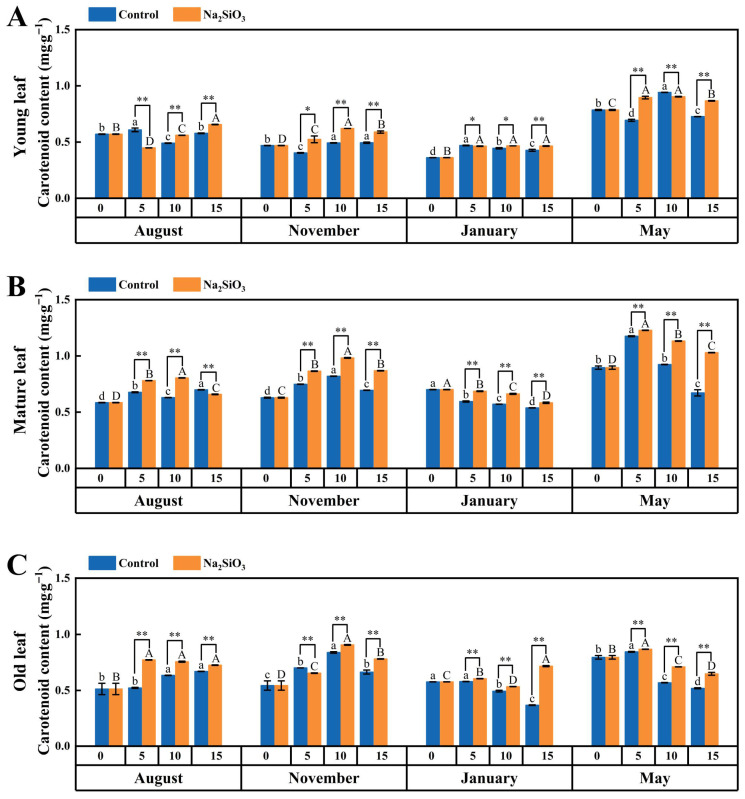
Carotenoid content in *D. brandisii* leaves at different phenological stages under control and addition of SS. (**A**) Young leaf. (**B**) Mature leaf. (**C**) Old leaf.

The carotenoid in *D. brandisii* leaves was high from the shooting stage until November, but decreased during the dormancy stage in January, and then significantly increased again in the branching and leafing stage in May (Figure 8).

After SS treatment, carotenoid levels in mature and old leaves of *D. brandisii* increased significantly on the 5th day in August, whereas no significant enhancement was observed in young leaves. In old leaves, this enhancement persisted until the 15th day. In November, carotenoid enhancement was more pronounced in mature leaves on the 5th day and after that its enhancement was significantly in *D. brandisii* leaves of all different ages. The carotenoid content was significantly improved in *D. brandisii* leaves of all different ages in January. The enhancement was more pronounced in mature and old leaves. Similarly to what was observed in January, the increase in carotenoid content was more evident in mature and old leaves, while in young leaves, a lower content was observed than in the control (Figure 8).

### 2.5. Photoassimilate Accumulation in D. brandisii Leaves Under Treatment at Different Phenological Stages

**Figure 9:**
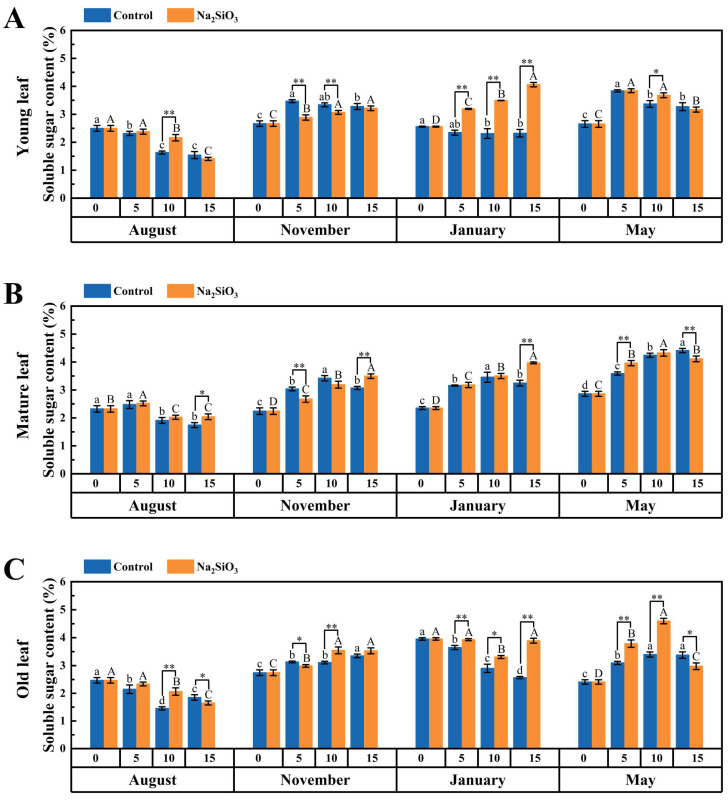
Soluble sugar content in *D. brandisii* leaves at different phenological stages under control and addition of SS. (**A**) Young leaf. (**B**) Mature leaf. (**C**) Old leaf.

The soluble sugar and starch represented the main photoassimilate accumulation. It was observed that the soluble sugar content in the young and mature leaves of *D. brandisii* increased continuously from August to November in the control group, with a significant decrease in the soluble sugar content of young leaves in dormancy stage in January, while the variation in the soluble sugar content of old leaves was not significant. During the branching and leafing stage in May, the soluble sugar content in both young and mature leaves increased significantly. Overall, the soluble sugar content in mature leaves was significantly higher than that in young and old leaves (Figure 9).

With SS treatment, the soluble sugar content in *D. brandisii* leaves at different phenological stages further improved. In August, the soluble sugar content in young and old leaves increased significantly on the 10th day, while the soluble sugar content in mature leaves did not increase significantly until the 15th day. In November, the soluble sugar content in the leaves treated with SS decreased significantly on the 5th day, but on the 10th day, the soluble sugar content in old leaves increased significantly, and on the 15th day, similar enhancement was observed in mature leaves. In January, the soluble sugar content in young and old leaves was improved significantly from the 5th day and continued to increase with time, while the soluble sugar content in mature leaves did not increase significantly until the 15th day. In May, the soluble sugar content in mature and old leaves further increased significantly on the 5th day. In contrast, the increase in young leaves was observed on the 10th day (Figure 9).

**Figure 10:**
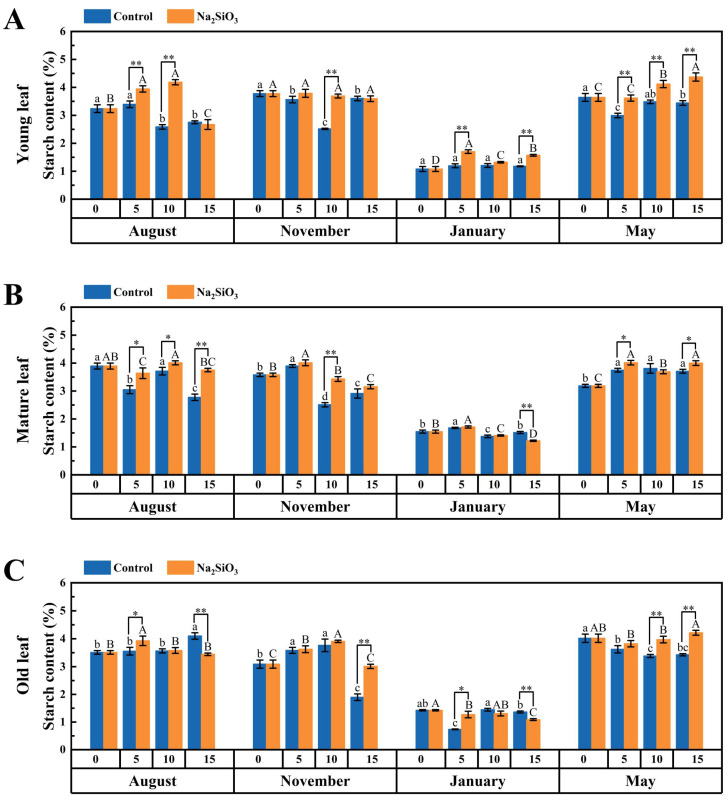
Starch content in *D. brandisii* leaves at different phenological stages under control and addition of SS. (**A**) Young leaf. (**B**) Mature leaf. (**C**) Old leaf.

It was observed that the starch content in *D. brandisii* leaves slightly increased from August to November, and then during the January of dormancy stage, it significantly decreased in contrast to the soluble sugar content, and in May during the branching and leafing stage, it significantly increased. The starch content in the leaves at different phenological stages was mature leaf > young leaf > old leaf (Figure 10).

With SS treatment, the starch content in *D. brandisii* leaves at different phenological stages increased. In August, the starch content in the leaves of different ages significantly further increased on the 5th day. The enhancement was more pronounced in young leaves on the 5th and 10th days, while it was particularly significant in mature leaves on the 15th day. In November, the starch enhancement in young and mature leaves was observed on the 10th day, while in old leaves, obvious enhancement was observed until the 15th day. The starch decreased in the dormancy stage in January. However, SS treatment promoted the starch synthesis, particularly in the young leaves, which showed significantly greater value than the control on the 5th and 15th day. In May, the starch content in young leaves showed greater value continuously with SS application compared to the control. The starch content in old leaves enhanced significantly starting on the 10th day (Figure 10).

### 2.6. Correlation Analysis

**Figure 11:**
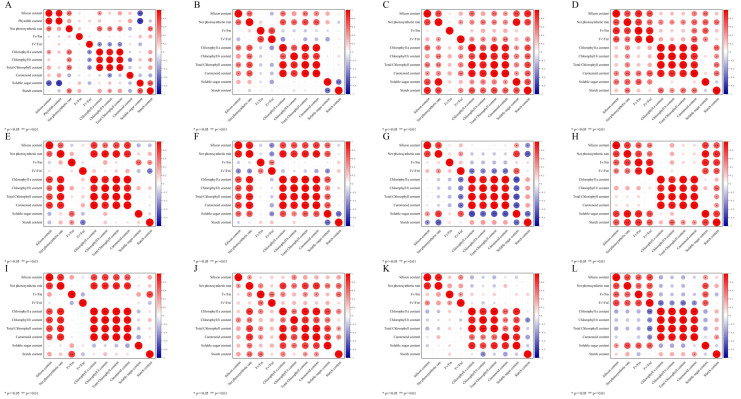
Correlation analysis of silicon and photosynthetic physiological indexes in *D. brandisii* leaves at different phenological stages. (**A**–**D**) Correlation analysis of silicon and photosynthetic physiological indexes in *D. brandisii* young leaves in August, November, January, and May. (**E**–**H**) Correlation analysis of silicon content and photosynthetic physiological indexes in *D. brandisii* mature leaves in August, November, January, and May. (**I**–**L**) Correlation analysis of silicon content and photosynthetic physiological indexes in *D. brandisii* old leaves in August, November, January, and May. One asterisk denoted significant differences at *p* < 0.05 and two asterisks at *p* < 0.01.

To further reveal the effects of silicon application at different phenological stages on the silicon accumulation and photosynthetic physiology in *D. brandisii* leaves, a correlation analysis among all indexes was performed (Figure 11).

Correlation analysis indicated that the silicon content in young leaves during the shooting stage in August had a significant positive correlation with net photosynthetic rate at the *p* ≤ 0.01 level (Figure 11A). The silicon content in mature and old leaves had a significant positive correlation with net photosynthetic rate, and photosynthetic pigments at the *p* ≤ 0.01 level (Figure 11E,I). This suggested that the accumulation of silicon in *D. brandisii* leaves during the shooting stage in August was closely related to net photosynthetic rate and photosynthetic pigments.

During the late shooting stage in November, the silicon content in *D. brandisii* young leaves showed a significant positive correlation with net photosynthetic rate, chlorophyll a, and total chlorophyll at the *p* ≤ 0.01 level (Figure 11B). The silicon content in mature leaves had a significant positive correlation with net photosynthetic rate, photosynthetic pigments, and soluble sugar content at the *p* ≤ 0.01 level (Figure 11F). In old leaves, the silicon content had a significant positive correlation with net photosynthetic rate and chlorophyll b at the *p* ≤ 0.05 level (Figure 11J). This indicated that the silicon accumulation during the late shooting stage of bamboo significantly affected the photosynthesis and played an important role in photosynthesis and the storage of photosynthetic assimilates.

During the dormancy stage in January, the silicon content in *D. brandisii* young leaves showed a significant positive correlation with the net photosynthetic rate and the content of soluble sugar and starch at the *p* ≤ 0.01 level (Figure 11C). The silicon content in mature leaves had a significant positive correlation with the net photosynthetic rate at the *p* ≤ 0.01 level (Figure 11G). Similarly, the silicon content in old leaves showed a significant positive correlation with the net photosynthetic rate at the *p* ≤ 0.01 level (Figure 11K). This indicated that the silicon accumulation during the dormant period of bamboo had much more significant impact on photosynthesis and photosynthetic assimilates in young leaves than in mature and old leaves.

During the branching and leafing stage in May, the silicon content in young leaves showed a significant positive correlation with the net photosynthetic rate, Fv/Fm, Fv′/Fm′, and soluble sugar content at the *p* ≤ 0.01 level (Figure 11D). In mature leaves, the silicon content was significantly positively correlated with the net photosynthetic rate, Fv/Fm, Fv′/Fm′, and photoassimilate accumulation (soluble sugar and starch content) at the *p* ≤ 0.01 level (Figure 11H). In old leaves, the silicon content was significantly positively correlated with the net photosynthetic rate, Fv/Fm, and Fv′/Fm′ at the *p* ≤ 0.01 level (Figure 11L). This indicated that the accumulation of silicon content in *D. brandisii* during the branching and leafing stage in May was closely related to photosynthesis and the storage of photoassimilates.

## 3. Discussion

### 3.1. Effect of SS on Silicon Content in D. brandisii Leaves at Different Phenological Stages

The accumulation of silicon in plants was closely related to phenological stages. Zhu et al. [17] reported that the silicon content in *Dendrocalamus giganteus* leaves increased from the branching and leafing stage to the late shooting stage, but decreased during the dormancy stage. This was consistent with the results from *D. brandisii* in this study. Moreover, silicon content in *D. brandisii* at different phenological stages showed an order of old leaf > mature leaf > young leaf. This was consistent with the previous research results that the silicon content of the young tissue was lower than that of the old tissue in bamboo [14,17,19]. This indicated that *D. brandisii* leaves continuously accumulated silicon, and there was a positive correlation between leaf age and silicon accumulation.

After SS application, the silicon content in the leaves of *D. brandisii* was further enhanced at various phenological stages. Zhang et al. [20] found that an appropriate exogenous silicon application promoted the absorption and utilization rate of silicon in the leaves of *D. brandisii*. August was the shooting stage of *D. brandisii*, the silicon content in young leaves was lower than in mature and old leaves, possibly because young leaves were in an active stage of cell division and differentiation, and the absorption and transportation channels for silicon had not fully matured yet. Leaves and roots are the main organs for bamboo to absorb silicon. After foliar SS application, the silicon content in young leaves significantly increased indicating that foliar silicon application was effective to promote the silicon absorption of young leaves. Silicic acid becomes concentrated and precipitated in the leaf cells via transpiration [21]. However, the silicon content in old leaves did not increase significantly as compared to the control, indicating the weak silicon absorption of old leaves. The temperature dropped in November, which was also the local dry season. Low temperatures and drought inhibited transpiration, slowing down the vertical transport of silicon in plants [22]. However, in November, the enhancement of silicon content in old leaves with SS application was particularly notable (increasing by 35–40% compared to the control). It was reported that the old leaves, as key organs for overwintering, reallocated silicon from senescent tissues to functional leaves, accumulating silicon to form silicified cell, which enhanced the mechanical strength of cell walls and increased resistance to stress [22]. The enhancement of silicon in *D. brandisii* leaves would help bamboo prepare for overwintering (Figure 1). In January, which was a cold winter season, the silicon content of *D. brandisii* leaves of different ages significantly increased, which was consistent with the results reported by Liu et al. [23] that silicon application can significantly increase the silicon content in cucumber leaves affected by cold. Habibi et al. [24] also reported similar results in corn leaves subjected to cold damage. May is the branching and leafing stage of bamboo. The silicon content in young leaves increased significantly, the reason of which might be that silicon was present in soluble form (Si(OH)_4_) in the cell, which may be related to the need for silicon to participate in the construction of the primary cell wall structure in rapidly growing cells [25].

### 3.2. Effect of SS on Photosynthesis in D. brandisii Leaves at Different Phenological Stages

Generally, *D. brandisii* experienced a stage of carbohydrate storage from the bamboo shooting stage in August to the late shooting stage in November. During this period, it was observed that the photosynthetic rate (Pn) and chlorophyll fluorescence rate of *D. brandisii* leaves maintained relatively high values which was consistent with more active photosynthesis of plants during the growing season [26]. After SS application, the Pn of *D. brandisii* leaves at different phenological stages was improved, especially during the branching and leafing stage (May). This may be due to the optimization of chloroplast structure by silicon, which increases the stability of photosynthetic proteins and improves stomatal regulation capacity, thereby effectively enhancing the activity of the plant photosynthetic system [26,27,28,29]. Previous studies have also reported that the application of silicon fertilizer enhances the growth of vegetables, such as Chinese cabbage and tomatoes, by increasing photosynthetic capacity and protecting the photosynthetic apparatus, thereby improving both growth performance and stress resistance [30,31]. However, despite the overall increasing trend in photosynthetic efficiency, there were differences in the response of leaves of different ages. In August and November, the enhancement of Pn in young leaves was greater, while in mature leaves it was pronounced in May. This may be related to the influence of SS on leaf structure and physiological characteristics at different stages of different leaf ages. The enhancement of photosynthetic activity in May and August helped *D. brandisii* maintain high photosynthetic efficiency, which was crucial for carbohydrate storage supporting shoot development, branching, and leaf formation. Due to the lower external temperatures in January, *D. brandisii* was in a dormant stage and its photosynthetic activity was accordingly low. The Pn of *D. brandisii* leaves treated with SS was significantly improved. This suggested that SS effectively improved photosynthesis during the dormancy stages, thereby enhancing the cold resistance of *D. brandisii*. The alleviation of extreme temperature stress in plants by silicon had also been proposed in previous reports [32]. Sodium silicate does not enhance photosynthesis by directly participating in the chemical steps of photosynthetic reactions. Instead, as a form of exogenous silicon (Si) supply, it triggers a series of cascading effects at the morphological, physiological, and molecular levels in plants [33,34]. Specifically, silicon enhances the stability of cell walls and cell membranes, maintains the integrity of chloroplast structures, and thereby improves the efficiency of photosystem II and the content of photosynthetic pigments. Additionally, silicon can optimize stomatal behavior, promote the absorption and supply of CO_2_, and reduce damage caused by reactive oxygen species (ROS) by enhancing the antioxidant system, thus contributing to a significant improvement in photosynthesis [32,35,36,37].

The Fv/Fm value is an important indicator for measuring the maximum photochemical efficiency of Photosystem II (PSII). It reflects the efficiency of PSII in converting light energy into chemical energy under dark-adapted conditions and is a key parameter for evaluating plant photosynthetic capacity and stress response [38]. Under light-adapted conditions, the change in ΦPSII is positively correlated with the change in Fv′/Fm′ value, indicating that under specific light conditions, the increase in PSII photochemical efficiency is consistent with the increase in its energy capture efficiency [39]. Nowakowska et al. [40] reported that silicon treatment enhanced the photosynthetic efficiency of European beech seedlings by reducing photoinhibition and increasing chlorophyll content, maintaining Fv/Fm values at a higher level. In this study, after applying SS to *D. brandisii*, the leaf chlorophyll fluorescence parameters (Fv/Fm and Fv′/Fm′) exhibited positive response during various phenological stages, especially in August and May, the positive impact of SS on chlorophyll fluorescence parameters was more pronounced. The Fv/Fm and Fv′/Fm′ of *D. brandisii* leaves in January was low, reflecting that the photosystem was inhibited under cold conditions. However, SS alleviated this inhibition to some extent.

Photosynthetic pigments are involved in the absorption and transfer of light energy during the photosynthetic CO_2_ assimilation process, including chlorophyll and carotenoids, which are crucial for plant photosynthesis [41,42]. Chlorophyll is a fundamental component of the photosynthetic mechanism, responsible for capturing, distributing, and converting light [43]. Carotenoids are a class of extremely important photosynthetic pigments in plant chloroplasts, mainly used for capturing light energy and transferring it to chlorophyll a for photosynthesis [44]. Silicon can be absorbed by plants and transported to chloroplasts, increasing chlorophyll content, enhancing photosynthetic efficiency, promoting gas exchange, and ensuring the effective utilization of CO_2_ in the process of carbon assimilation [45,46]. The application of exogenous silicon promoted the formation of chloroplast thylakoid membranes, which was beneficial for the synthesis of plant chlorophyll, maintaining a higher level of chlorophyll, and promoting plant growth and development [47]. Muneer et al. [48] showed that the addition of silicon increased the content of photosynthetic pigments, net photosynthesis, transpiration rate, and stomatal conductance, and improved the cytochrome b6/f and ATP synthase complex under salt stress. In this study, SS application to *D. brandisii* at different phenological stages promoted the photosynthetic pigments, particularly in August and May, where a significant increase in chlorophyll content was observed in both young and mature leaves, indicating that SS had a significant promoting effect on chlorophyll synthesis during shooting stage and branching and leafing stage. However, in January during the dormant stage, a significant decrease in overall chlorophyll content was observed, which may be due to the low-temperature environment exacerbating the degradation of chloroplasts and chlorophyll, as well as reducing the activity of enzymes involved in synthesis within the cells, thereby decreasing chlorophyll synthesis [49]. After SS treatment, the chlorophyll content in *D. brandisii* leaves increased to some extent; however, the effect gradually weakened over time, indicating that the influence of SS was limited under the low temperatures of January. Carotenoids in *D. brandisii* leaves significantly increased after treatment with SS, indicating that SS was conducive to promoting the synthesis of carotenoids. The carotenoids enhancement in dormancy stage in January was also significant, suggesting that carotenoids might help *D. brandisii* to clear reactive oxygen species accumulated due to low-temperature stress, reduced the peroxidation of lipid membranes by reactive oxygen species and the toxicity to photosynthetic organs, maintained the stability of membrane morphology, and enhanced its cold resistance [50].

### 3.3. Effect of SS on Photoassimilate Accumulation in D. brandisii Leaves at Different Phenological Stages

Soluble sugars and starch are key products of plant photosynthesis and the main forms of carbohydrate storage in nutritive tissues [51]. Sugars are crucial for plant growth and development, acting as metabolic intermediates that are resynthesized into other substances through metabolic pathways, such as structural materials of cells, storage substances, and the carbon skeletons of amino acids [52,53,54]. Starch is the primary carbohydrate reserve in plants and plays a critical role in their metabolism, growth, and development [55]. Soluble sugar is the substrate for starch synthesis and there is a close relationship between the soluble sugar content and starch synthesis in plants. Starch accumulation occurs when the carbohydrate supply exceeds demand [51].

Silicon enhances photosynthetic efficiency of leaves, increasing the accumulation of photosynthetic products, thereby indirectly increasing the content of soluble sugars [56]. Silicon promotes the synthesis and conversion of soluble sugars by enhancing the activity of key enzymes related to the metabolism of soluble sugars in citrus (phosphofructokinase and hexokinase) [57]. Foliar application of silicon effectively improved the photosynthetic efficiency and lodging resistance of soybeans under normal light and shade conditions, and by regulating carbon metabolism, it promoted starch accumulation, thereby increasing yield and quality of soybeans [58]. In this study, with SS treatment, the soluble sugar and starch in *D. brandisii* leaves at various phenological stages also enhanced.

In August, the soluble sugar content in *D. brandisii* leaves was relatively low due to the consumption for shoot bud development. After SS treatment, the soluble sugar in young and old leaves increased on the 10th day, promoting sugar synthesis to support shoot bud development. During this time, the starch accumulation in *D. brandisii* leaves of different ages enhanced significantly on the 5th day and continuous enhancement was observed with time of application, suggesting that SS enhanced photosynthesis, leading to a carbohydrate surplus. The promotion of photosynthesis (Pn, Fv/Fm and Fv′/Fm′) in *D. brandisii* after SS treatment in August further supports this phenomenon.

Sugars also play a protective role against oxidative stress by regulating antioxidant enzyme activity and scavenging free radicals [59]. In this study, SS treatment in January significantly increased soluble sugar content in *D. brandisii* young leaves, enhancing cold resistance and stress tolerance. This was further supported by the enhanced starch content, which was critical for bamboo’s survival during the cold season and vernal growth.

In May, SS application significantly increased starch content in *D. brandisii* young and mature leaves, promoting photosynthesis and sugar synthesis to support branching, leaf formation, and shoot bud differentiation. This suggested that SS not only improves photosynthetic efficiency but may also provide more sufficient energy support for bamboo by regulating carbon assimilation and subsequent metabolic pathways.

Correlation analysis further revealed the linkage mechanism of silicon-photosynthesis-carbon metabolism in *D. brandisii* leaves at different phenological stages. After applying SS, a significant positive correlation between silicon content and photosynthetic physiological indicators was observed in *D. brandisii* leaves (Figure 11). In August, the silicon content showed a significant positive correlation with Pn and chlorophyll *a* (Figure 11A,E), indicating that silicon might optimize the light environment (reducing UV damage, uniform light distribution), indirectly protecting the stability of chlorophyll *a* and photosynthetic efficiency [60]. Although the silicon content in young leaves was relatively low, silicon still significantly affected their transmission spectra, indicating that silicon may play a key role in developing leaf photosynthesis and stress resistance [60]. In this study, the silicon content in young leaves showed a significant correlation with photosynthetic performance, which further supports this view. After the bamboo shooting stage in November, as the temperature dropped in autumn and seasonal drought occured, the silicon content in young leaves was significantly positively correlated with chlorophyll. Silicon enhances mechanical strength through deposition in plants and forms a physical barrier, which effectively improves plants’ resistance to pathogens and overall disease resistance [61,62]. Silicon application on tomato leaves protected chloroplast structure and function, and maintained high photosynthetic efficiency by reducing the accumulation of reactive oxygen species (ROS) and malondialdehyde (MDA) in chloroplasts [63]. This indicates that silicified cells may indirectly maintain chloroplast integrity by providing mechanical protection (such as reducing pathogen infection or physical damage), delaying chlorophyll degradation. When *D. brandisii* entered the dormancy stage in January, its physiological activities significantly decreased. However, during this period, the silicon content in *D. brandisii* leaves was significantly positively correlated with the soluble sugar content, revealing that silicon may maintain cellular osmotic balance and alleviate stress under adverse conditions. The significant positive correlation between silicon content and Fv/Fm during the branching and leafing stage in May (Figure 11D,H,L) reflected the role of silicon in protecting the photosystem, with the most significant effect in old leaves, indicating that silicon may delay the aging of the photosystem.

We inferred that the role of silicon accumulation in *D. brandisii* leaves of different ages was multifaceted. Young leaves rely on siliconized structures and silicon’s resistance, mature leaves focused on the photosynthetic enhancement and metabolic synergy of silicon, and old leaves utilized silicon’s aging and function delay. These results collectively indicated that SS treatment, through multi-pathway synergistic regulation, combined with different phenological stages and leaf developmental phases, maximized the synergistic effect of silicon in photosynthetic efficiency and stress resistance, providing a theoretical basis for the precise application of silicon fertilizer in bamboo forest cultivation.

Although this study reveals the effects of foliar sodium silicate application on the silicon accumulation and photosynthesis of *D. brandisii* leaves at different phenological stages, there are still some problems worthy of further discussion. For instance, although sodium silicate exerts a similar promotional effect on leaf photosynthesis as silicon itself, the potential influence of sodium ions on photosynthesis cannot be ruled out. Additionally, this study has limitations in terms of its duration, and the impact of factors such as climate variability should also be considered. In the future, molecular biological approaches can be combined to further analyze the expression patterns of silicon transport genes during phenological stages to deepen the understanding of the silicon-photosynthesis coupling mechanism.

## 4. Materials and Methods

### 4.1. Sample Collection and Preparation

The experiment was conducted in cultivated bamboo garden of Southwest Forestry University, Kunming, P. R. China. Six clusters of *D. brandisii* were selected. Each cluster contained at least three bamboo culms of each age class (1, 2, ≥3 years). According to the previous literature on the vegetative phenological stages of sympodial bamboo [64], the vegetative phenological stages of *D. brandisii* during the whole year were recorded and described in Table 1. On 1 August 2022 (Shooting stage), 1 November 2022 (Late shooting stage) and 1 January 2023 (Dormancy stage), 1 May 2023 (Branching and leafing stage), three clusters were sprayed with 2 mmol/L sodium silicate (SS) solution, the concentration of which was based on the effects of exogenous silicon application on *D. brandisii* in the previous literature [20]. Another three clumps were sprayed with distilled water to be the control. Ten pieces of young, mature and old leaves of the culms of each age class (1, 2, ≥3 years) in each cluster were separately obtained on the 5th day, 10th day and 15th day. Three replicates were set for each stage. The collected leaves were divided into two parts equally, one was put into an ultra-low temperature refrigerator for measuring physiological indicators, the other was put into the oven for 110 °C for 30 min, turned to 60 °C to dry until constant weight, and stored for silicon determination.

### 4.2. Experimental Methods

#### 4.2.1. Determination of Silicon Content

Samples were dried to constant weight at 60 °C in the oven and then ground in a Wiley mill. Ground materials were passed through a no.40 mesh sieve shaker, but retained on no.60 mesh and were used for the determination of silicon content. The ash content was determined using the Chinese national standard GB/T 2677.3–93 [65]. Then, following the standard for silicon GB/T7978 [66], 5 mL of 6 mol/L HCl was added to a crucible containing ash content and evaporated in a electric furnace. After three repetitions, the residue was rinsed with distilled water and filtered through quantitative filter paper. It was then washed with hot water until no chloride ions were detected in the wash solution. The residue along with the filter paper was transferred to a pre-weighed porcelain crucible (constant mass). The crucible was placed in a muffle furnace and heated at 575 ± 25 °C for 2 h. Silicon content was subsequently determined by gravimetric measurement.

#### 4.2.2. Determination of Net Photosynthetic Rate (Pn)

The net photosynthetic rate (Pn) was measured between 9:00 am and 10:00 am before sampling using Portable Photosynthesis System (produced by Zhejiang Top Cloud-Agri Technology Co., Ltd. (Hangzhou, China) Model: 3051D). At least nine intact leaves of each type (young, mature, and old) were selected from each bamboo cluster for measurement.

#### 4.2.3. Determination of Chlorophyll Fluorescence Rate

The chlorophyll fluorescence rate of *D. brandisii* leaf samples was determined by Yaxin-1162 Chlorophyll Fluorescence Meter (produced by ansatech Instruments Co., Ltd. ( Norfolk, England ) Model: Handy PEA) following the method from Maxwell and Johnson [67] and Genty et al. [68]. At least nine intact leaves of young, mature and old, respectively, of each bamboo cluster were selected to conduct with clip-on light and without clip-on light between 10:00 am and 11:00 am.

#### 4.2.4. Determination of Photosynthetic Pigment

The chlorophyll and carotenoid concentrations in the leaves were determined by extracting the pigments with 96% ethanol, quartz sand, and calcium carbonate powder and measuring the absorbance at 470 nm, 649 nm, and 665 nm using a spectrophotometer (UV-5500, Metash, Shanghai, China) [69]. The contents of chlorophyll *a*, chlorophyll *b*, and carotenoid were determined according to the method described by Lichtenthaler [70].

#### 4.2.5. Determination of Soluble Sugar and Starch Content

The soluble sugar and starch content were determined using the phenol–sulfuric acid method according to Glassop et al. [71] and Dubois et al. [72]. The absorbance was measured at 485 nm using a UV spectrophotometer (UV-4500, MWTASH, Shanghai, China). Each sample was tested in triplicate.

#### 4.2.6. Statistical Analysis

The means derived from the experiments were statistically analyzed using multiple comparisons using a one-way ANOVA and independent sample *t*-tests. The least significant difference method (LSD) to determine the level of significance at *p* ≤ 0.05. These analyses were conducted using SPSS (Statistical Package for the Social Sciences) 20.0 for Windows software (SPSS Inc., Chicago, IL, USA).

## 5. Conclusions

In summary, this study demonstrated that foliar application of SS had a significant impact on the silicon content, photosynthesis, and the accumulation of photoassimilate in *D. brandisii* leaves at different phenological stages. SS significantly increased the silicon content in *D. brandisii* leaves, showing a significant correlation. August and May were critical periods for the silicon accumulation. SS treatment significantly improved the net photosynthetic rate (Pn), chlorophyll content, and photosystem activity (Fv/Fm, Fv′/Fm′), especially during the bamboo shooting stage in August and the branching and leafing stage in May.

Correlation analysis showed that silicon content was significantly positively correlated with photosynthetic parameters (Pn, chlorophyll a/b) and the accumulation of photoassimilate (soluble sugar, starch), confirming that silicon optimized leaf light capture and carbon assimilation capacity. Young leaves at the shooting stage (August) of *D. brandisii* responded the fastest to silicon absorption from SS, while mature leaves at the branching and leafing stage (May) showed the most significant photosynthetic enhancement. The differences in how *D. brandisii* leaves of different ages respond to SS at various phenological stages identify a critical time window for the precise application of silicon fertilizer. These results elucidated the synergistic mechanism of silicon in the accumulation of silicon and photosynthetic function in *D. brandisii*, providing a theoretical basis for the efficient use of silicon fertilizer in bamboo forest cultivation.

## Figures and Tables

**Table 1:** Phenological characteristics of *Dendrocalamus brandisii.*

Sample Timing	Phenological Stage	Phenology of Bamboo Clump
August 2022 Summer/rainy season	Shooting	Shoot buds continued forming and developing. Shoots generated out of the ground constantly.
November 2022 Autumn/dry season	Late shooting	Shooting completed, and most finished height growth.
January 2023 Winter/dry season	Dormancy	New culms ceased growing and the bamboo clumps entered dormancy.
May 2023 Spring/dry season	Branching and leafing	Branches and leaves extended simultaneously, and shoot buds started sprouting at the bases of 1- and 2-year culms.

## Data Availability

The datasets generated during and/or analyzed during the study are available from the corresponding author upon reasonable request.
